# Comparison of the predictive value of four insulin resistance surrogates for the prevalence of hypertension: a population-based study

**DOI:** 10.1186/s13098-022-00907-9

**Published:** 2022-09-26

**Authors:** Wenke Cheng, Fanliang Kong, Siwei Chen

**Affiliations:** 1 Medical Faculty, University of Leipzig, Leipzig, Germany; 2 Department of Cardiology and Pneumology, University Medical Center of Göttingen, Georg-August University, Göttingen, Germany; 3 German Centre for Cardiovascular Research (DZHK), Partner Site, Göttingen, Germany; 4 Department of Cardiovascular Medicine, The Third Hospital of Nanchang, No.1268, Jiuzhou Street, Chaoyang New District, Nanchang, Jiangxi China

**Keywords:** Insulin resistance, Triglyceride-glucose index, Triglycerides/high-density lipoprotein cholesterol ratio, TyG-BMI, METS-IR, Bayesian network

## Abstract

**Background:**

Several studies have investigated the association of insulin resistance (IR) surrogates and the risk of hypertension. However, it is unclear whether there exist differences between different IR surrogates and hypertension risk. Therefore, this study aimed to explore the association of four IR surrogates (triglyceride-glucose index (TyG index), triglyceride-glucose index with body mass index (TyG-BMI), triglycerides/high-density lipoprotein cholesterol ratio (TG/HDL-c), and metabolic score for IR (METS-IR)) with the prevalence of hypertension.

**Methods:**

This is a cross-sectional study with a total of 117,056 participants. Data were extracted from a computerized database established by Rich Healthcare Group in China, which included all medical records of participants who received a health check-up from 2010 to 2016. IR surrogates were grouped into quartiles as continuous variables, and multivariate logistic regression was performed to estimate the association between different IR surrogate levels and the prevalence of hypertension. Results were expressed as odds ratios (ORs) and 95% confidence intervals (CIs). Missing data were accounted by multiple imputation. These analyses were considered as the sensitivity analysis. Meanwhile, the Bayesian network (BN) model was constructed to further evaluate the relationship between baseline characteristics and the four IR surrogates and the prevalence of hypertension, as well as the importance of every single variable for the prevalence of hypertension.

**Results:**

Multivariate logistic regression analysis revealed that TyG-BMI and METS-IR were independent risk factors for the prevalence of hypertension that increased significantly with increasing TyG-BMI and METS-IR (p for trend < 0.001). The area under the TyG-BMI curve (AUC) was 0.681 [95% CI: 0.677–0.685], and the cut-off value was 199.5, with a sensitivity and specificity of 65.57% and 61.18%, respectively. While the area under the METS-IR curve (AUC) was 0.679 [95% CI: 0.674–0.683], and the cut-off value was 33.61, with a sensitivity and specificity of 69.67% and 56.67%, respectively. The BN model presented that among these four IR surrogates and related variables, TyG-BMI was the most important predictor of hypertension prevalence, with a significance of 34%. The results before and after multiple imputation were similar.

**Conclusion:**

TyG-BMI and METS-IR were independent risk factors for the prevalence of hypertension. TyG-BMI and METS-IR had good predictive value for the prevalence of hypertension, and TyG-BMI was superior to METS-IR.

**Supplementary Information:**

The online version contains supplementary material available at 10.1186/s13098-022-00907-9.

## Background

Hypertension which ranked as the third leading cause of disability-adjusted life years [1] is a critical public health challenge worldwide. The prevalence of hypertension is expected to increase globally, especially in developing countries [2]. By 2025, the number of adults with hypertension will reach 1.56 billion in the world, and one-third of adults will suffer from hypertension in China [3, 4]. In this context, early identification of specific populations at potential risk of developing hypertension is essential for lowering disability and mortality associated with hypertension.

Clinically, hypertension patients are frequently observed to coexist with type 2 diabetes mellitus (T2DM), and it is hypothesized that insulin resistance (IR) is a common pathophysiological aspect of T2DM and hypertension [5]. Moreover, substantial evidence suggests that IR plays a vital role in the development of hypertension [6, 7], suggesting that IR may serve as an adjunctive tool to identify individuals at risk for hypertension. The hyperinsulinemic–euglycemic clamp test is considered to be the “gold standard” method for evaluating insulin sensitivity [8]. However, due to its complexity, invasiveness, and high cost, the hyperinsulinemic–euglycemic clamp test is currently not suitable for routine clinical practice. Therefore, a simpler and more practical method of indicating IR is required. Previously, several clinical studies have assessed individual IR levels by several non-insulin-based fasting IR indicators, namely, IR surrogates, including the triglyceride-glucose (TyG) index [9], TyG index with body mass index (TyG-BMI) [10], triglycerides/high-density lipoprotein cholesterol ratio (TG/HDL-c) [11], and metabolic score for IR (METS-IR) [12]. IR surrogates are calculated from lipid parameters and related indexes. To some extent, their discoveries have addressed the difficulties in identifying IR patients.

Recently, several studies have investigated the association of IR surrogates and the risk of hypertension [5, 6, 13, 14], and they have consistently revealed that higher IR surrogates were strongly associated with the risk of hypertension. However, it is unclear whether there are differences between different IR surrogates and hypertension risk. Therefore, a cross-sectional study with a large sample that involved four IR surrogates (TyG, TyG-BMI, TG/HDL-c, and METS-IR) was conducted, aiming to explore the association of these four IR surrogates with the prevalence of hypertension.

## Methods

### Study design and data extraction

This is a secondary study, and data was extracted from a computerized database established by Rich Healthcare Group in China, where all medical records of participants who received a health check-up from 2010 to 2016 were incorporated. The detailed design and main results have been previously published [15]. Specifically, totally 211,833 adults were recruited from 32 health screening centers in 11 Chinese cities (Shanghai, Beijing, Nanjing, Suzhou, Shenzhen, Changzhou, Chengdu, Guangzhou, Hefei, Nantong, and Wuhan). Moreover, all participants completed a detailed questionnaire, including demographics, lifestyle, and family history of chronic diseases, during their first visit to the health screening center.

Participants’ clinical characteristics, including weight, height, and blood pressure, were measured by the trained staff. Fasting venous blood samples were collected after fasting for at least 10 h. In terms of biochemical parameters, including total cholesterol (TC), high-density lipoprotein cholesterol (HDL-c), low-density lipoprotein cholesterol (LDL-c), serum creatinine (SCr), fasting blood glucose (FPG), blood urea nitrogen (BUN), alanine aminotransferase (ALT), and aspartate aminotransferase (AST), they were measured by the uniform automated analyzer (Beckman 5800). Additionally, BMI was calculated by dividing weight by the square of height. All data were collected under the uniform and standardized procedure.

The original study was approved by the rich healthcare group review board. The data were anonymous; the baseline information was retrieved retrospectively, and the rich healthcare group review board waived the requirement for informed consent [16]. This study complies with *the Declaration of Helsinki*.

### Study population

**Fig. 1:**
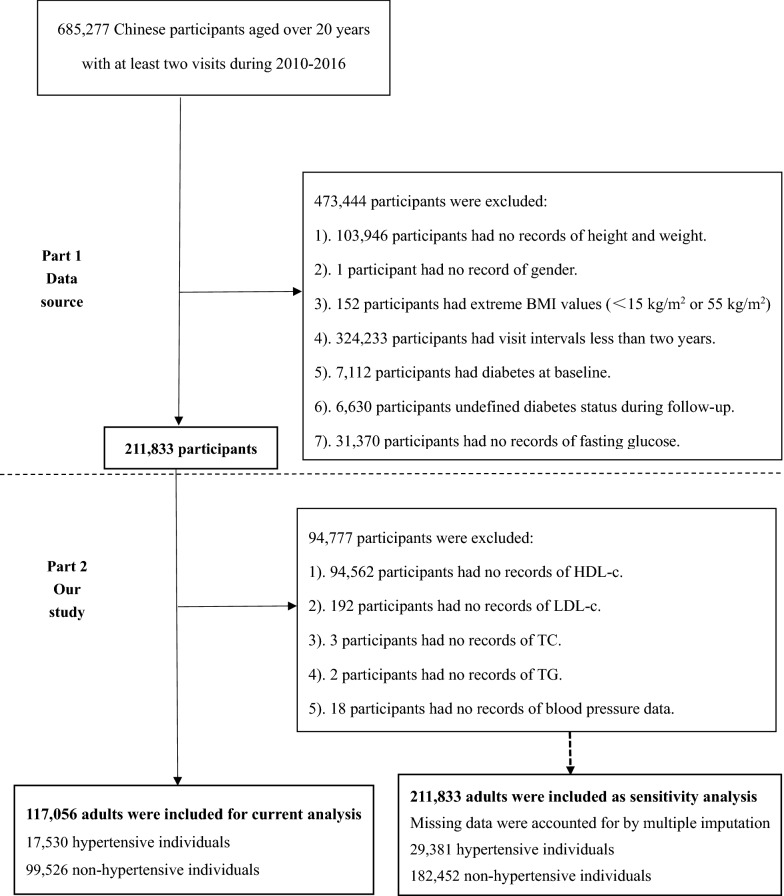
Study flow

Consistent with the initial study, the subjects were non-diabetic and aged 20–99 years, and did not develop diabetes during the 2-year follow-up between 2010 and 2016. As shown in Fig. 1, the flow chart of the study population selection consisted of two parts. The first part is the selection process for the initial study. Which included 211,833 participants [15], while the second part is the selection process, which further excluded 94,777 participants for the following specific reasons: (1) 94,562 participants did not have HDL-c on record; (2) 192 participants had no record of LDL-c; (3) 3 participants had no record of TC; (4) 2 participants had no record of TG; (5) 18 participants had no record of blood pressure data. Finally, 117,056 Chinese adults featuring 17,530 hypertension and 99,526 non-hypertension participants were involved in this study for analysis.

### Definitions

The exposure of interests was four IR surrogates, namely, TyG index, TyG-BMI, TG/HDL-c, and METS-IR. They were calculated by the following formula: TyG index = ln[TG(mg/dl) *FPG(mg/dl)/2] [5]; TyG-BMI = TyG*BMI [6]; TG/HDL-c = TG divided by HDL-c [5]; METS-IR = Ln[(2*FPG) + TG]*BMI)/(Ln[HDLc]) [5]. Age was divided into 3 groups, < 45 (young), 45–60 (middle-aged), and ≥ 60 years (old). With reference to the current Chinese guidelines [17], the body mass index was classified into 3 groups, namely < 24 kg/m^2^ (normal/underweight), 24–28 kg/m^2^ (overweight), and ≥ 28 kg/m^2^ (obesity). Meanwhile, AST and ALT were divided into < 40 U/L (normal) and ≥ 40 U/L (abnormal) [18]. All participants were required to rest in a quiet environment for at least 5 min before blood pressure measurement. Blood pressures were obtained by the trained staff with a standard mercury sphygmomanometer based on office blood pressure measurements. Due to the special circumstances of physical examination, most participants cannot do multiple blood pressure detection on different days. As a result, the blood pressure value of all participants was the initial one-time blood pressure level. According to the Chinese guidelines for the prevention and control of hypertension (2018 edition) [19], participants were grouped into hypertensive and non-hypertensive groups. Hypertension was defined as individuals with resting systolic blood pressure (SBP) ≥ 140 mmHg or diastolic blood pressure (DBP) ≥ 90 mmHg, whereas non-hypertension was defined as individuals with SBP < 140 mmHg and DBP < 90 mmHg. Hypertension was further divided into three groups: Class I (SBP 140–159 or DBP 90–99 mmHg), Class II (SBP 160–179 or DBP 99–109 mmHg) and Class III (SBP ≥ 180 or DBP ≥ 110 mmHg).

### Statistical analysis

Due to the skewed distribution of the data, continuous variables were expressed as median and interquartile range (IQR), and dichotomous variables were denoted as numbers (percentages). Differences between dichotomous variables were analyzed by the Chi-square test, while the Man-Whitney test was used to analyze the differences in continuous variables for two groups, and Kruskal-Wallis one-way analysis of variance (ANOVA) and Dunn’s test would be adopted for multiple comparisons. Spearman’s correlation coefficient was employed to assess the correlation between four IR surrogates and the SBP or DBP, whereas subgroup analysis were performed by stratifying for age, sex, BMI, ALT, and AST. Furthermore, IR surrogates were grouped into quartiles as continuous variables, and multivariate logistic regression was performed to estimate the association between different IR surrogate levels and the prevalence of hypertension, with results expressed as odds ratios (ORs) and 95% confidence intervals (CIs), while p for trend would be obtained. Among the 211,833 participants, data on lipid and blood pressure levels were missing in 94,777 participants. To lower the bias caused by population selection, multiple imputation was conducted for missing data. Multiple imputation was performed based on the predictive mean matching (PMM) algorithm, 10 replications and Markov chain Monte Carlo (MCMC) methods. Then, the optimal set of datasets was selected according to the alpha-cronbach factor for sensitivity analysis. All statistical analysis was done using SPSS (version 26; SPSS Inc., Chicago, IL, USA) and GraphPad (version 9.0; USA, San Diego, CA). A two-tailed *p* < 0.05 was considered statistically significant.

To further evaluate the relationship between baseline characteristics and the four IR surrogates and the prevalence of hypertension, as well as the importance of every single variable for the prevalence of hypertension, a Bayesian network (BN) model was constructed. The BN model typically combines probability theory and graph theory, and is one of the probabilistic graphical models that reveal probabilistic dependencies between variables (nodes), which can be used to show the probability relationship between one disease and its related factors [20, 21]. The model was established based on the tree augmented native (TAN) algorithm in the model section of SPSS Modeler 18.0, and the parameter learning method was selected as Bayesian adjustment of small cell counts [22]. Arrows connecting the two nodes indicate that these two random variables are causally or unconditionally independent; if two nodes have no arrows, then the random variables are conditionally independent [20]. Moreover, each circle in the BN model represents a predictor whose shade of color refers to its importance for the occurrence of hypertension, with darker colors indicating higher importance.

## Results

The median age of the overall population was 41 (IQR 34–53) years, with hypertensive individuals (17,530) accounting for 15% of the overall population. As shown in Table 1, among the baseline characteristics of participants, age, percentage of current smokers and drinkers, SBP, DBP, BMI, FPG, ALT, AST, BUN, SCr, TG, TC, and LDL were higher in the hypertensive population than in the non-hypertensive population. However, the proportion of females and family history of diabetes, and HDL-C were lower in the hypertensive population. In the comparison of the four IR surrogates, the values were higher in the hypertensive population. Except for the difference in TG between the two groups, the differences in baseline characteristics between the two populations before and after multiple imputation were found to be in consistence (Additional file 1: Table S1).

**Table 1:** Baseline information of the overall population

	Total (n = 117,056)	Non-hypertension (n = 99,526)	Hypertension (n = 17,530)	*p* value
Age (years)	41 (34–53)	39 (33–50)	53 (41–63)	< 0.001
Female (%)	54,099 (46.2)	48,304 (48.5)	5795 (33.1)	< 0.001
Current smoker (%)	6674 (5.7)	5,520 (5.5)	1154 (6.6)	< 0.001
Current drinker (%)	872 (0.7)	651 (0.6)	221 (1.3)	< 0.001
Family history of diabetes (%)	2651 (2.3)	2,367 (2.4)	284 (1.6)	< 0.001
SBP (mmHg)	118 (107–130)	115 (106–124)	144 (138–153)	< 0.001
DBP (mmHg)	73 (66–81)	72 (65–78)	91 (84–95)	< 0.001
BMI (kg/m^2^)	23.2 (21–25.5)	22.9 (20.8–25.1)	25.1 (23–27.3)	< 0.001
FPG (mmol/L)	5.0 (4.61–5.35)	4.95 (4.6–5.3)	5.19 (4.8–5.62)	< 0.001
ALT (U/L)	18.2 (13–27.8)	18 (12.9–26.8)	22 (15.7–33)	< 0.001
AST (U/L)	22.0 (18.7–26.9)	21.8 (18.2–26)	24.2 (20.7–29.7)	< 0.001
BUN (mmol/L)	4.57 (3.85–5.4)	4.53(3.81–5.35)	4.78(4.06–5.63)	< 0.001
SCr (μmol/L)	71.3 (59.3–83.0)	70.5(58.8–82.4)	75.8(64.23–86.0)	< 0.001
TC (mg/dl)	181.70 (159.67–204.90)	179.77 (158.51–202.97)	191.75 (168.56–216.50)	< 0.001
TG (mg/dl)	97.46 (67.34–146.19)	97.46 (67.34–146.19)	97.46 (67.34–147.08)	0.772
LDL (mg/dl)	104.38 (88.53–121.78)	103.22 (87.76–120.62)	110.95 (93.94–128.74)	< 0.001
HDL-c (mg/dl)	51.80 (45.23–59.92)	52.19 (45.23–59.92)	51.42 (44.46–59.15)	< 0.001
Lipid Profile				
TG/HDL-c	1.90 (1.27–2.93)	1.89 (1.26–2.91)	1.95 (1.30–2.99)	< 0.001
TyG index	8.37 (7.99–8.79)	8.37 (7.99–8.78)	8.41 (8.03–8.82)	< 0.001
TyG-BMI	193.87 (173.52–216.06)	190.87 (171.30–212.31)	211.46 (191.42–233.13)	< 0.001
METS-IR	33.21 (29.37–37.44)	32.63 (28.95–36.76)	36.48 (32.79–40.56)	< 0.001

Continuous data are expressed as median (interquartile range) due to the skewed distribution. The p-value is a comparison between the normotension and hypertension groups

*FPG* fasting plasma glucose, *TG* triglycerides, *TC* total cholesterol, *HDL-c* high-density lipoprotein cholesterol, *LDL* low-density lipoprotein cholesterol, *SCr* serum creatinine, *BUN* blood urea nitrogen, *ALT* alanine aminotransferase, *AST* aspartate aminotransferase, *BMI* Body mass index, *TG/HDL-c* triglycerides/high-density lipoprotein cholesterol ratio, *TyG index* Triglyceride-glucose index, *TyG-BMI* TyG index with body mass index, *METS-IR* metabolic score for insulin resistance, *SBP* Systolic blood pressure, *DBP* Diastolic blood pressure

### Correlation between SBP or DBP and the four IR surrogates

Spearman correlation analyses suggested that SBP was positively correlated with TgG index (r = 0.047, p < 0.001), TyG-BMI (r = 0.344, p < 0.0001), TG/HDL-c (r = 0.037, p < 0.001) and METS-IR (r = 0.349, p < 0.0001). Similarly, DBP was positively related to TgG (r = 0.036, p < 0.0001), TyG-BMI (r = 0.32, p < 0.0001), TG/HDL-c (r = 0.046, p < 0.0001) and METS-IR (r = 0.33, p < 0.0001).

### Subgroup analysis of the four IR surrogates between the non-hypertensive and hypertensive groups

Stratified analysis was performed according to sex, age, AST, ALT, and BMI, as displayed in Table 2. Regardless of gender, AST and ALT, TyG index, TyG-BMI, and METS-IR were significantly higher in the hypertensive group, whereas TG/HDL-c was higher only in the female hypertensive group and in the hypertensive group with normal liver function (p < 0.05). Similarly, compared with the non-hypertensive group, TyG, TyG-BMI and METS-IR were noticeably increased in the hypertensive group regardless of age level, whereas TG/HDL-c was not significantly different in middle-aged adults (p = 0.094). Additionally, TyG-BMI and METS-IR were higher in the hypertensive group than in the non-hypertensive group at different BMI levels (p < 0.001) to a great extent, and TG/HDL-c and TyG index differed in different subgroups.

**Table 2:** Subgroup analysis of four IR surrogates differences between non-hypertensive and hypertensive groups

	Non-hypertension	Hypertension	*p*-value	Non-hypertension	Hypertension	*p*-value
Gender	Male			Female		
TG/HDL-c	2.02 (1.35–3.12)	2.01 (1.34–3.08)	0.104	1.76 (1.18–2.70)	1.83 (1.23–2.79)	< 0.001
TyG index	8.37 (7.99–8.79)	8.41 (8.03–8.83)	< 0.001	8.36 (7.98–8.77)	8.42 (8.04–8.82)	< 0.001
TyG-BMI	200.29 (181.34–220.96)	214.94 (195.43–236.06)	< 0.001	180.81 (164.14–200.30)	204.27 (183.97–226.61)	< 0.001
METS-IR	34.87 (31.35–38.67)	37.35 (33.76–41.28)	< 0.001	30.31 (27.4–33.88)	34.63 (30.94–38.64)	< 0.001
AST	< 40 U/L			≥ 40 U/L		
TG/HDL-c	1.88 (1.26–2.87)	1.92 (1.29–2.94)	0.005	1.97 (1.32–3.05)	2.04 (1.36–3.13)	0.274
TyG index	8.37 (7.99–8.78)	8.43 (8.05–8.83)	< 0.001	8.39 (8.0–8.8)	8.45 (8.09–8.87)	0.006
TyG-BMI	190.99 (171.66–211.84)	210.27 (190.74–231.11)	< 0.001	211.3 (189.7–233.62)	231.67 (207.55–256.37)	< 0.001
METS-IR	32.65 (29.09–36.57)	36.15 (32.59–40.07)	< 0.001	36.71 (32.49–41.04)	39.86 (35.95–44.41)	< 0.001
ALT	< 40 U/L			≥ 40 U/L		
TG/HDL-c	1.87 (1.25–2.88)	1.92 (1.29–2.95)	< 0.001	2.06 (1.38–3.15)	2.06 (1.39–3.16)	0.720
TyG index	8.36 (7.98–8.78)	8.41 (8.03–8.83)	< 0.001	8.39 (8.0–8.80)	8.42 (8.07–8.81)	0.002
TyG-BMI	188.44 (169.63–208.89)	208.01 (188.70–228.93)	< 0.001	213.98 (194.32–234.96)	229.28 (209.32–251.46)	< 0.001
METS-IR	32.13 (28.65–36.04)	35.82 (32.20–39.66)	< 0.001	37.55 (33.76–41.53)	40.04 (36.3–44.5)	< 0.001

BMI	< 24 kg/m2			24–28 kg/m2			≥ 28 kg/m2		
	non-Hypertension	Hypertension	*p*-value	non-Hypertension	Hypertension	*p*-value	non-Hypertension	Hypertension	*p*-value
TG/HDL-c	1.81 (1.21–2.79)	1.84 (1.23–2.82)	0.221	2.02 (1.35–3.12)	2 (1.34–3.07)	0.101	2.08 (1.39–3.23)	2.04 (1.36–3.13)	0.037
TyG index	8.35 (7.97–8.77)	8.4 (8.01–8.81)	< 0.001	8.4 (8.0–8.81)	8.42 (8.05–8.84)	< 0.001	8.4 (8.03–8.81)	8.42 (8.05–8.82)	0.136
TyG-BMI	177.08 (163.1–190.9)	185 (172.4–197.1)	< 0.001	214 (205.6–229.6)	217.2 (205.6–229.6)	< 0.001	248.82 (235.2–264.7)	251.7 (238–268.7)	< 0.001
METS-IR	30 (27.45–32.49)	31.54 (29.21–33.6)	< 0.001	37.2 (35.2–39.5)	37.5 (35.5–39.8)	< 0.001	43.59 (41.22–46.68)	43.99 (41.3–47)	0.001

Age	≤ 44 year			45–59 year			≥ 60 year		
	non-Hypertension	Hypertension	*p*-value	non-Hypertension	Hypertension	*p*-value	non-Hypertension	Hypertension	*p*-value
TG/HDL-c	1.88 (1.26–2.90)	1.99 (1.33–3.04)	< 0.001	1.92 (1.27–2.95)	1.94 (1.30–3.0)	0.094	1.87 (1.26–2.89)	1.93 (1.3–2.94)	< 0.001
TyG index	8.35 (7.97–8.76)	8.37 (8.0–8.79)	0.001	8.4 (8.01–8.81)	8.41 (8.05–8.83)	0.007	8.41 (8.03–8.83)	8.45 (8.06–8.85)	0.001
TyG-BMI	186.3 (167.15–208.1)	213.5 (191.1–238)	< 0.001	197.3 (179.2–217.3)	212.7 (192–233.6)	< 0.001	200.1 (181.58–229.6)	208.9 (190–229.6)	< 0.001
METS-IR	31.79 (29.23–36.04)	37 (32.83–41.64)	< 0.001	33.78 (30.36–37.58)	36.62 (33.08–40.5)	< 0.001	34.31 (30.71–38.08)	36 (32.4–39.79)	< 0.001

*IR* insulin resistance,*TG* triglyceride, *HDL-c* high-density lipoprotein cholestero, *ALT* alanine aminotransferas, *AST* aspartate aminotransferas, *BMI* Body mass inde, *TG/HDL-c* triglycerides/high-density lipoprotein cholesterol rati, *TyG index* Triglyceride-glucose inde, *TyG-BMI* TyG index with body mass inde, *METS-IR* metabolic score for insulin resistance

### Multivariate logistic regression analyses of the four IR surrogates and the prevalence of hypertension

TG/HDL-c, TyG index, TyG-BMI, and METS-IR were taken as continuous variables divided into quartiles, with the first quartile (Q1) as the reference group, as shown in Table 3. In the crude model, no covariates were adjusted; age and gender were adjusted in Model 1; all characteristics in the baseline were fully adjusted in model 2. In the crude model, the prevalence of hypertension increased dramatically with increasing levels of TG/HDL-c, TyG index, TyG-BMI, and METS-IR (p for trend < 0.001). However, after adjusting for age and sex, the prevalence of hypertension did not differ observably at different levels of TG/HDL-C (p for trend = 0.152). Furthermore, the positive correlation between TyG-BMI and METS-IR and the prevalence of hypertension remained when fully adjusted for the maximum variables (p for trend < 0.001). Consistent with the results before multiple imputation, we also observed an independent positive association of TyG-BMI and METS-IR with the prevalence of hypertension in the sensitivity analysis. This association was also shown in TyG index and TG/HDL (Additional file 1: Table S2).

**Table 3:** Multivariable logistic regression of four IR surrogates and the prevalence of hypertension

	Crude model		Model 1		Model 2	
	OR (95% CI)	*p-*value	OR (95% CI)	*p-*value	OR (95% CI)	*p-*value
TG/HDL-c						
Q 1 (≤ 1.27)	Ref.		Ref.		Ref.	
Q 2 (1.27–1.90)	1.069 (1.021–1.119)	< 0.001	1.034 (0.985–1.086)	0.181	1.012 (0.938–1.092)	0.759
Q 3 (1.90–2.93)	1.096 (1.047–1.147)	< 0.001	1.034 (0.985–1.086)	0.175	0.991 (0.914–1.075)	0.825
Q 4 (≥ 2.93)	1.124 (1.074–1.176)	< 0.001	1.038 (0.989–1.090)	0.128	0.952 (0.854–1.060)	0.369
*p* for trend	< 0.001		0.152		0.413	
TyG index						
Q 1 (≤ 7.99)	Ref.		Ref.		Ref.	
Q 2 (7.99–8.37)	1.10 (1.051–1.154)	< 0.001	1.068 (1.016–1.122)	0.01	0.997 (0.905–1.056)	0.977
Q 3 (8.37–8.79)	1.175 (1.122–1.231)	< 0.001	1.090 (1.038–1.145)	< 0.001	0.995 (0.918–1.077)	0.995
Q 4 (≥ 8.79)	1.230 (1.175–1.288)	< 0.001	1.106 (1.054–1.162)	< 0.001	0.929 (0.838–1.030)	0.929
*p* for trend	< 0.001		< 0.001		0.319	
TyG-BMI						
Q 1 (≤ 173.52)	Ref.		Ref.		Ref.	
Q 2 (173.52–193.87)	1.975 (1.857–2.10)	< 0.001	1.512 (1.418–1.612)	< 0.001	1.174 (1.059–1.302)	0.002
Q 3 (193.87–216.06)	3.264 (3.080–3.458)	< 0.001	2.163 (2.035–2.229)	< 0.001	1.320 (1.176–1.483)	< 0.001
Q 4 (≥ 216.06)	5.863 (5.546–6.198)	< 0.001	3.724 (3.511–3.950)	< 0.001	1.404 (1.202–1.642)	< 0.001
*p* for trend	< 0.001		< 0.001		< 0.001	
METS-IR						
Q 1 (≤ 29.37)	Ref.		Ref.		Ref.	
Q 2 (29.37–33.21)	2.507 (1.934–2.188)	< 0.001	1.596 (1.497–1.703)	< 0.001	1.267 (1.136–1.414)	< 0.001
Q 3 (33.21–37.44)	3.520 (3.322–3.731)	< 0.001	2.357 (2.216–2.506)	< 0.001	1.489 (1.307–1.696)	< 0.001
Q 4 (≥ 37.44)	5.841 (5.523–6.178)	< 0.001	3.776 (3.555–4.011)	< 0.001	1.510 (1.262–1.807)	< 0.001
*p* for trend	< 0.001		< 0.001		< 0.001	

*OR* odds ratio, *CI* confidence interval, *Q* quartile, *IR* insulin resistance, *TG/HDL-c* triglycerides/high-density lipoprotein cholesterol ratio, *TyG index* Triglyceride-glucose index, *TyG-BMI* TyG index with body mass index, *METS-IR* metabolic score for insulin resistance

Model 1 adjust age and gender

Model 2 adjust Model 1 + BMI (kg/m^2^), FPG (mmol/L), ALT(U/L), AST(U/L), BUN, Scr, smoking status (current smoker or not), drinking status (current drinker or not), family history of diabetes (Yes or No)

### Predictive value of four IR surrogates for the prevalence of hypertension

As shown in Table 4, TyG-BMI and METS-IR had a good predictive value for the prevalence of hypertension. The predictive value of TyG-BMI for the prevalence of hypertension was mildly preferable to METS-IR. The area under the TyG-BMI curve (AUC) was 0.681 [95% CI: 0.677–0.685] and the cut-off value was 199.5, with a sensitivity and specificity of 65.57% and 61.18%, respectively. Whereas, the area under the METS-IR curve (AUC) was 0.679 [95% CI: 0.674–0.683] and the cut-off value was 33.61, with a sensitivity and specificity of 69.67% and 56.67%, respectively. Despite attempts to combine TyG-BMI and METS-IR to further predict the prevalence of hypertension, the predictive value did not improve. However, when TyG-BMI, METS-IR were combined with age, the predictive value of the prevalence of hypertension increased to 77.4% and 77.3%, respectively. In consistence with the results before multiple imputation, the predictive values of TyG-BMI and METS-IR for the prevalence of hypertension remained higher than that of TyG index and TG/HDL (Additional file 1: Table S3).

**Table 4:** Predictive value of four IR substitutes for the prevalence of hypertension

	AUC	95% CI	Cut-off Point	Sensitivity (%)	Specificity (%)
TyG index	0.524	0.519–0.528	8.19	65.96	38.09
TyG-BMI	0.681	0.677–0.685	199.5	65.57	61.18
TG/HDL-c	0.512	0.508–0.517	1.975	49.11	52.89
METS-IR	0.679	0.674–0.683	33.61	69.67	56.67
TyG-BMI + Age	0.774	0.771–0.778	0.118*	78.8	62.3
METS-IR + Age	0.773	0.769–0.776	0.119*	78.5	62.3

*TG/HDL-c* triglycerides/high-density lipoprotein cholesterol ratio, *TyG index* Triglyceride-glucose index, *TyG-BMI* TyG index with body mass index, *METS-IR* metabolic score for insulin resistance, *AUC* area under the curve

*refers to the predicted probability calculated by logistic regression

### Differences in four IR surrogates for different blood pressure levels

**Fig. 2:**
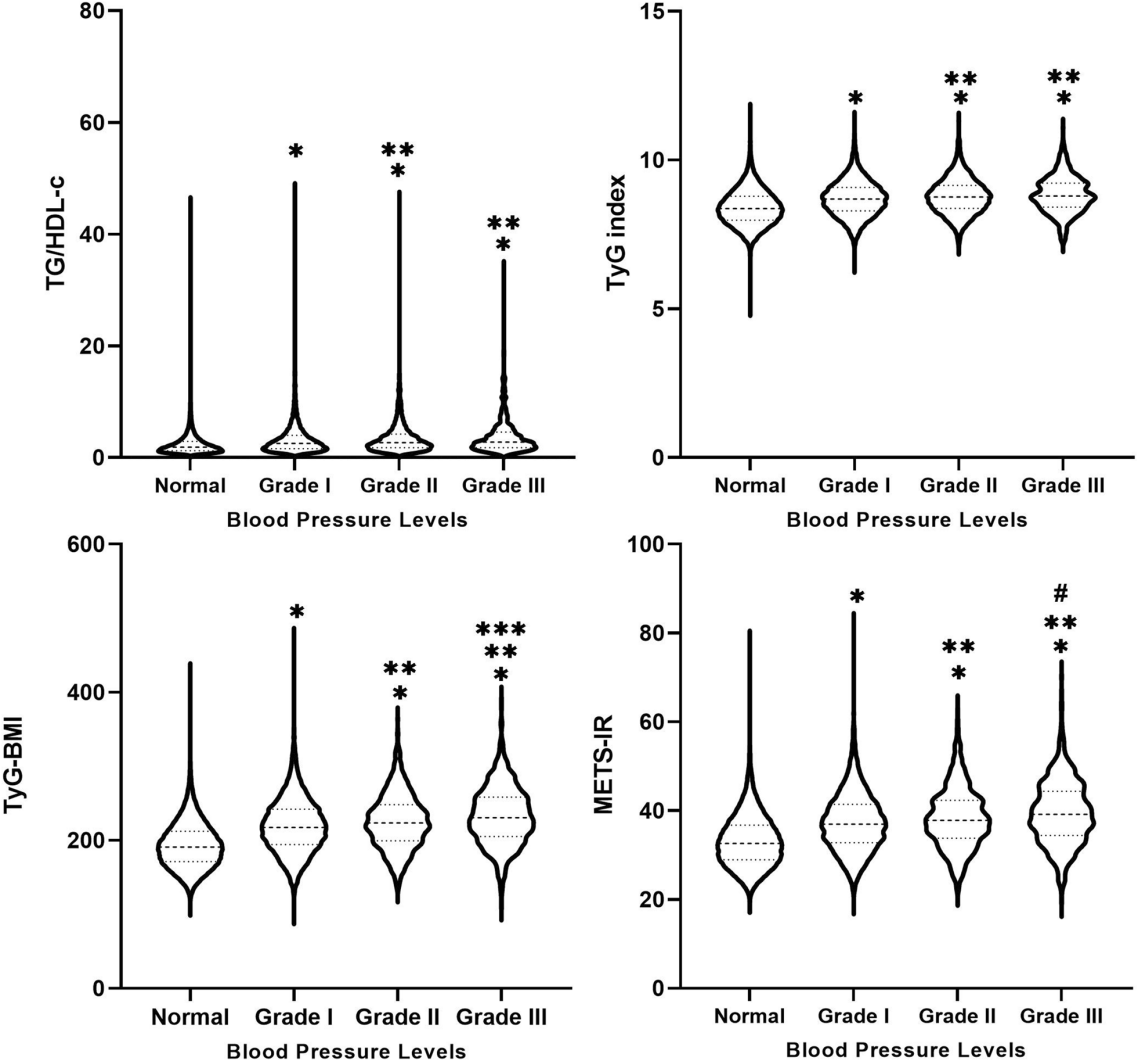
Between-group differences in four IR surrogates at different blood pressure levels. *Indicates Grade I, Grade II and Grade III compared with normal group with p-value < 0.001. **Indicates Grade II and Grade III compared with Grade I with p-value < 0.001. ***Indicates Grade III compared with Grade II with p-value < 0.001. ^#^Indicates Grade III compared with Grade II with p-value < 0.05

To further analyze the intergroup differences of the four IR surrogates at different blood pressure levels, the hypertensive group was further divided into Grades I-III. As shown in Fig. 2, the levels of the 4 IR surrogates were higher in the Grades I-III group than in the normal group, and higher in the Grade II and Grade III groups than in the Grade I group (p < 0.001). Moreover, TyG-BMI and METS-IR were higher in the Grade III group than in the Grade II group (p < 0.05).

### BN model analysis of the relationship between each predictor on the prevalence of hypertension

**Fig. 3:**
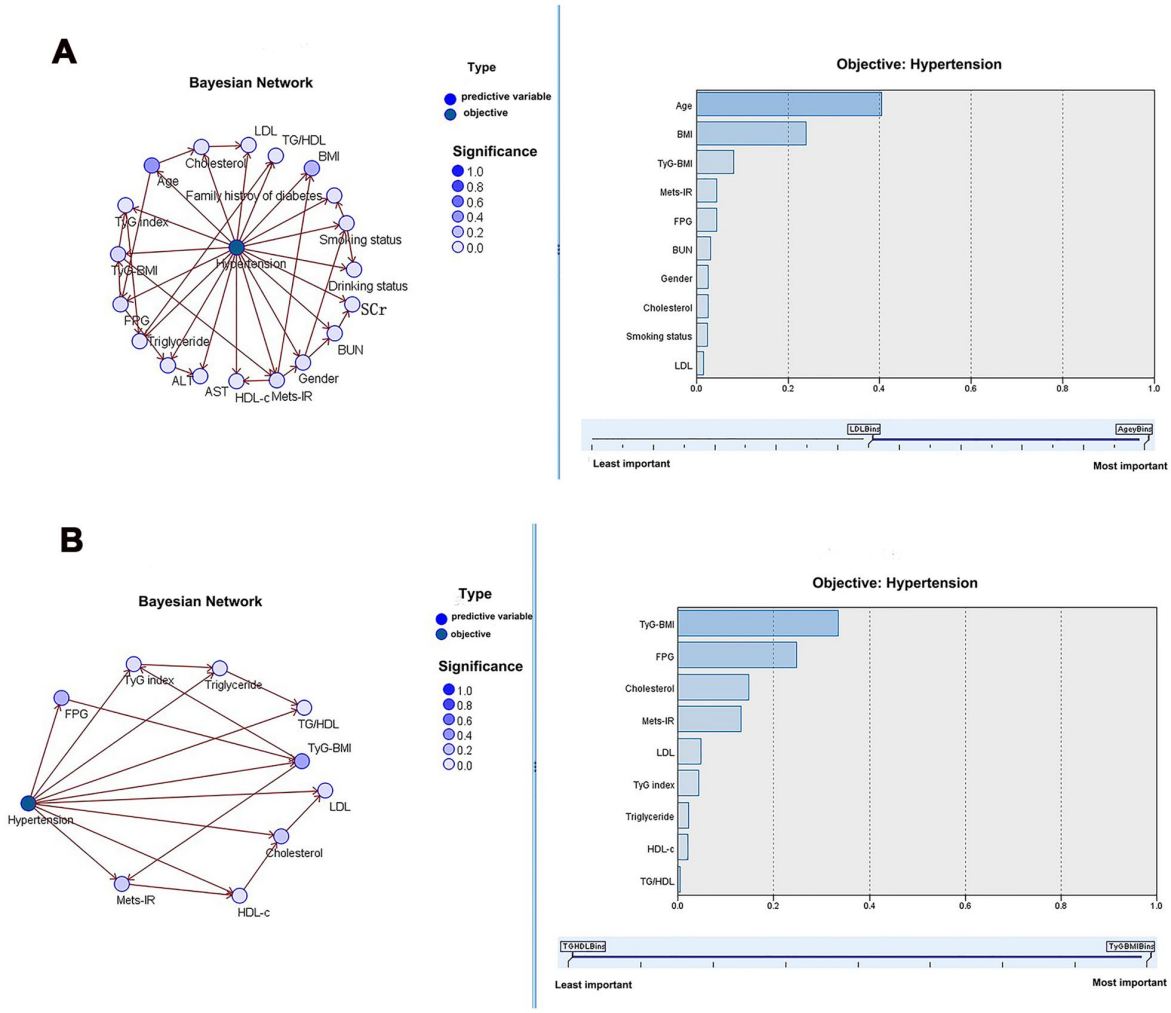
**A** Bayesian network model of hypertension prevalence and all clinical characteristics. **B** Bayesian network model of hypertension prevalence and four IR surrogates-related characteristics

The BN model including four IR surrogates and all baseline variables showed that age directly influenced cholesterol and FPG, and TyG-BMI directly affected TyG index and METS-IR. Among all variables in Fig. 3A, age was the most significant predictor for the prevalence of hypertension with an importance of 41%. Similarly, the BN model containing four IR surrogates and related variables indicated that TyG-BMI had a direct influence on TyG index and METS-IR, and TyG index indirectly influenced TG/HDL-c, as presented in Fig. 3B. Moreover, among these four IR surrogates and related variables, TyG-BMI was the most significant predictor for the prevalence of hypertension with an importance of 34%. The results of BN models before and after multiple imputation were similar (Additional file 1: Fig. S1).

## Discussion

This is a large-scale cross-sectional study, and the findings could be summarized as follows: (1) TG/HDL-c, TyG index, TyG-BMI and METS-IR were significantly increased in the hypertensive group compared to the non-hypertensive group. (2) TyG-BMI and METS-IR were independently and positively associated with the prevalence of hypertension. (3) TyG-BMI and METS-IR had good predictive value for the prevalence of hypertension, and TyG-BMI was superior to METS-IR.

Hypertension is a leading risk factor for death in China, with 254,000 deaths in 2017, mainly due to cardiovascular disease [23]. The diagnosis of hypertension is simple, but early identification of hypertension is still very difficult in China, because for most people, hypertension is featured with no clinical symptoms at the early stage and is often ignored. Currently, the prevalence of hypertension among adults in China is reported to be 23.2% (244.5 million), while the awareness rate is 46.9, and the control rate is only 15.3% [24]. IR surrogates are reproducible and inexpensive indicators that can be readily collected in primary care and clinical routine management. Moreover, in this survey, it is emphasized that TyG-BMI and METS-IR are independent risk factors for the prevalence of hypertension, and have good predictive value for hypertension in people without diabetes, providing a new approach for the preliminary screening of hypertension.

Several previous studies have investigated the relationship between IR surrogates and hypertension, while their results have not been fully consistent due to differences in study populations and observed endpoints. Bala C et al. pointed out that TG/HDL-C and TyG-BMI were independently associated with the prevalence of hypertension in the study based on 2,124 Romanian people [25]. Li YX et al. held that TyG index, TyG-BMI, TG/HDL-C and METS-IR were independently connected with the prevalence of hypertension based on 4,352 community residents aged > 65 years, while TyG-BMI and METS-IR were good predictors of the prevalence of hypertension (AUC = 0.63), which is similar to our findings [6]. Jian S et al. found that elevated TyG index was significantly related to high risk of hypertension but not to isolated diastolic hypertension in middle-aged and older adults [13]. Although there were differences in the results of these studies, they all consistently presented a strong association of IR surrogates with hypertension. As for the differences in the study results, there were the following reasons. Firstly, the study population was a non-diabetic population, and the age range of the study population was 20–99 years, including young, middle-aged, and older adults. Although the results were more generalizable, it was observed from the BN model that age, as a very important factor influencing the prevalence of hypertension, may attenuate the effect of IR surrogates on hypertension. Secondly, our study was based on 11 multi-centers with a total of over 200,000 participants, and the results of the study were more robust and more broadly generalizable than those of previous single-center studies.

IR surrogates are calculated from lipid parameters. Thus, the association between IR surrogates and hypertension can be partially explained by lipid parameters. Dyslipidemia and hypertension are traditional risk factors for cardiovascular disease and are strongly associated with the onset of cardiovascular disease. Previous studies have supported the hypothesis of a biological interrelationship between blood pressure and lipids [26, 27], and the increased risk of hypertension with dyslipidemia has been further confirmed in cohort studies [28, 29]. Although the ACC/AHA and ESC/EAS guidelines have recommended LDL-C as the most important lipid risk factor and therapeutic target for cardiovascular disease [30], lipid levels are susceptible to multiple factors including genetics, lifestyle, certain disorders, and medications [31]. Our previous study showed that HDL-C levels did not decrease to a great extent in the hypertensive population, but rather increased, considering that the “dysfunctional” HDL population represents a certain proportion of the real world [32]. In fact, HDL-c levels are not truly reflective of HDL-c function, as demonstrated in several previous studies [33, 34]. IR surrogates are an emerging independent predictor of hypertension, and higher IR surrogates are strongly associated with the presence of hypertension, even when LDL-C or HDL-C is well controlled [14].

## Future perspectives

Combining multiple risk factors to predict the risk of hypertension is the future direction of precision medicine. We first apply the BN model to infer the importance of each predictor on the prevalence of hypertension, which helps to understand which risk factors are primary and which are secondary. Meanwhile, single risk factor exerts a limited effect on the presence of hypertension. In this study, TyG-BMI and METS-IR alone predicted hypertension prevalence at around 68%, and when combined with age, the predictive value increased to 77%, significantly higher than traditional risk factors (< 70%) [35]. Furthermore, although there existed differences in the relationship between TyG index, TG/HDL-c and hypertension prevalence before and after multiple imputation (in terms of independent correlations), the predictive value of TyG-BMI and METS-IR for hypertension prevalence was significantly better than the former.

## Limitations

Firstly, the study design was cross-sectional, and the causal relationship between IR surrogates and risk of hypertension could not be clarified. Meanwhile, the absence of other patient data, such as diet, lifestyle, body fat distribution, and other laboratory indicators such as homocysteine, could not further assess the impact of these aspects on the results. Secondly, the participants in this paper were those of the community health check-up. The subjects were relatively healthy, and the diagnosis of hypertension depended on the baseline blood pressure level. The current prevalence of hypertension might be underestimated as the raw data lacked patients’ previous history of hypertension and antihypertensive medication use. The above may generate inaccurate interpretation of the results, and we will consider designing our study to improve this deficiency in the future. Finally, the study population was a non-diabetic population, and the results of this study might not be generalized to the diabetic population. During the complete case analysis, some participants were excluded from the analysis due to missing data. Although we performed the multiple imputation to maximize statistical power, and the results before and after imputation were very similar, selection bias may still exist.

## Conclusion

Among TG/HDL-c, TyG index, TyG-BMI and METS-IR, TyG-BMI and METS-IR were independent risk factors for the prevalence of hypertension. TyG-BMI and METS-IR had good predictive value for the prevalence of hypertension, and TyG-BMI was superior to METS-IR.

## Supplementary Information

**Additional file1:Table S1.** Baseline information of the overall population after multiple imputation *.**Table S2.** Multivariable logistic regression of four IR surrogates and the prevalence of hypertension after multiple imputation. **Table S3.** Predictive value of four IR substitutes for the prevalence of hypertension after multiple imputation. **Figure S1**. Bayesian network model of hypertension prevalence and all clinical characteristics after multiple imputation. **Figure S2.** Bayesian network model of hypertension prevalence and four IR surrogates-related characteristics after multiple imputation.

## Data Availability

The raw data is available (https://datadryad.org/stash/dataset/doi: 10.5061%2Fdryad.ft8750v).
