# Supplementary material for: Comparison of the predictive value of four insulin resistance surrogates for the prevalence of hypertension: a population-based study
Source: Diabetol Metab Syndr. 2022 Sep 26;14:137. doi: 10.1186/s13098-022-00907-9 (PMC9511744; doi:10.1186/s13098-022-00907-9)

| ***Supplementary Table 1*. Baseline information of the overall population after multiple imputation *** | | | | |
| --- | --- | --- | --- | --- |
|  | Total  (n=211,833) | non-Hypertension  (n=182,452) | Hypertension  (n=29,381) | *p* value |
| Age(years) | 38 (32-50) | 51(38-62) | 37(32-47) | < 0.001 |
| Female (%) | 95,710 (45.2) | 8969(30.5) | 86741(47.5) | < 0.001 |
| Current smoker (%) | 12,075 (5.7) | 1985(6.8) | 10090(5.5) | < 0.001 |
| Current drinker (%) | 1,351 (0.6) | 335(1.1) | 1016(0.6) | < 0.001 |
| Family history of diabetes (%) | 4,344 (2.1) | 444(1.5) | 3900(2.1) | < 0.001 |
| SBP (mmHg) | 118 (107-130) | 144(138-153) | 115(106-125) | < 0.001 |
| DBP (mmHg) | 73 (66-81) | 90(83-95) | 72(65-78) | < 0.001 |
| BMI (kg/m^2^) | 23 (20.8-25.4) | 25.1(22.9-27.4) | 22.7(20.6-25.0) | < 0.001 |
| FPG (mmol/L) | 4.92 (4.56-5.30) | 5.14(4.75-5.58) | 4.89(4.5-5.24) | < 0.001 |
| ALT (U/L) | 18 (13-28) | 23(16-34) | 17.6(12.6-26.8) | < 0.001 |
| AST (U/L) | 22 (18.6-26.7) | 24.1(20.5-30) | 21.6 (18.1-26.0) | < 0.001 |
| BUN (mmol/L) | 4.56 (3.83-5.41) | 4.8(4.06-5.69) | 4.52(3.80-5.37) | < 0.001 |
| SCr (μmol/L) | 70.9 (59-82) | 75(64-85) | 70(58.6-81.4) | < 0.001 |
| TC (mg/dl) | 177.84 (157.35-201.81) | 189.43(166.24-214.18) | 177.06(155.41-199.87) | < 0.001 |
| TG (mg/dl) | 94.8 (64.68-141.76) | 126.7(88.6-126.7) | 88.6(62.02-133.79) | < 0.001 |
| LDL (mg/dl) | 102.45 (86.60-119.85) | 109.02(92.4-127.19) | 101.29(86.21-118.30) | < 0.001 |
| HDL-c (mg/dl) | 51.80 (44.85-59.92) | 51.03(43.69-58.76) | 51.8(45.23-59.23) | < 0.001 |
| **Lipid Profile** |  |  |  |  |
| TG/HDL-c | 1.81 (1.19-2.90) | 2.47(1.61-3.95) | 1.72(1.15-2.72) | < 0.001 |
| TyG index | 8.33 (7.94-8.77) | 8.68(8.27-9.09) | 8.27(7.90-8.70) | < 0.001 |
| TyG-BMI | 192.11 (167.61-219.73) | 218.8(194.25-243.64) | 187.75(164.90-214.22) | < 0.001 |
| METS-IR | 32.87 (28.85-37.58) | 37.16(32.91-41.6) | 32.21(28.41-36.71) | < 0.001 |

*Continuous data are expressed as median (interquartile range) due to the skewed distribution. The p-value is a comparison between the normotension and hypertension groups.

FPG, fasting plasma glucose. TG, triglycerides. TC, total cholesterol. HDL-c, high-density lipoprotein cholesterol. LDL, low-density lipoprotein cholesterol. SCr, serum creatinine. BUN, blood urea nitrogen. ALT, alanine aminotransferase. AST, aspartate aminotransferase. BMI, Body mass index. TG/HDL-c, triglycerides/high-density lipoprotein cholesterol ratio. TyG index, Triglyceride-glucose index. TyG-BMI, TyG index with body mass index. METS-IR, metabolic score for insulin resistance. SBP, Systolic blood pressure. DBP, Diastolic blood pressure.

| ***Supplementary Table 2*. Multivariable logistic regression of four IR surrogates and the prevalence of hypertension after multiple imputation** | | | | | | |
| --- | --- | --- | --- | --- | --- | --- |
|  | Crude Model |  | Model 1 |  | Model 2 |  |
|  | OR (95% CI) | *p-*value | OR (95% CI) | *p-*value | OR (95% CI) | *p-*value |
| TG/HDL-c |  |  |  |  |  |  |
| Q 1 (≤1.19) | Ref |  | Ref |  | Ref |  |
| Q 2 (1.19-1.81) | 1.766 (1.689-1.848) | < 0.001 | 1.394(1.330-1.461) | < 0.001 | 1.005(0.848-1.191) | 0.952 |
| Q 3 (1.81-2.90) | 2.775(2.660-2.894) | < 0.001 | 1.850(1.770-1.935) | < 0.001 | 1.219(1.038-1.432) | 0.016 |
| Q 4 (≥2.90) | 4.506(4.328-4.692) | < 0.001 | 2.604(2.494-2.719) | < 0.001 | 1.523(1.298-1.787) | < 0.001 |
| *p* for trend | < 0.001 |  | < 0.001 |  | < 0.001 |  |
| TyG index |  |  |  |  |  |  |
| Q 1 (≤7.94) | Ref |  | Ref |  | Ref |  |
| Q 2 (7.94-8.33) | 1.914(1.824-2.007) |  | 1.448(1.378-1.522) | < 0.001 | 1.396(1.160-1.680) | < 0.001 |
| Q 3 (8.33-8.77) | 3.288(3.144-3.438) |  | 1.989(1.898-2.084) | < 0.001 | 1.395(1.165-1.670) | < 0.001 |
| Q 4 (≥8.77) | 5.793(5.551-6.407) |  | 2.999(2.866-3.139) | < 0.001 | 1.953(1.630-2.340) | < 0.001 |
| *p* for trend | < 0.001 |  | < 0.001 |  | < 0.001 |  |
| TyG-BMI |  |  |  |  |  |  |
| Q 1 (≤167.61) | Ref |  | Ref |  | Ref |  |
| Q 2 (167.61-192.11) | 2.372(2.248-2.503) | < 0.001 | 1.705(1.613-1.802) | < 0.001 | 1.518(1.218-1.892) | < 0.001 |
| Q 3 (192.11-219.73) | 4.741(4.510-4.985) | < 0.001 | 2.729(2.590-2.876) | < 0.001 | 1.822(1.445-2.296) | < 0.001 |
| Q 4 (≥219.73) | 9.525(9.078-9.995) | < 0.001 | 5.239(4.979-5.512) | < 0.001 | 2.456(1.866-3.233) | < 0.001 |
| *p* for trend | < 0.001 |  | < 0.001 |  | < 0.001 |  |
| METS-IR |  |  |  |  |  |  |
| Q 1 (≤28.85) | Ref |  | Ref |  | Ref |  |
| Q 2 (28.85-32.87) | 2.210(2.100-2.325) |  | 1.691(1.604-1.782) |  | 1.234(1.008-1.512) | 0.042 |
| Q 3 (32.87-37.58) | 4.143(3.951-4.344) |  | 2.622(2.495-2.756) |  | 1.418(1.146-1.754) | 0.001 |
| Q 4 (≥37.58) | 7.976(7.622-8.347) |  | 4.780(4.555-5.017) |  | 1.747(1.356-2.250) | < 0.001 |
| *p* for trend | < 0.001 |  | < 0.001 |  | < 0.001 |  |

OR, odds ratio. CI, confidence interval. Q, quartile. IR, insulin resistance. TG/HDL-c, triglycerides/high-density lipoprotein cholesterol ratio. TyG index, Triglyceride-glucose index. TyG-BMI, TyG index with body mass index. METS-IR, metabolic score for insulin resistance.

Model 1 adjust age and gender.

Model 2 adjust Model 1+ BMI (kg/m^2^), FPG (mmol/L), ALT(U/L), AST(U/L), BUN, Scr, king status (current smoker or not), drinking status (current drinker or not), family history of diabetes (Yes or No).

| ***Supplementary table 3*. Predictive value of four IR substitutes for the prevalence of hypertension after multiple imputation** | | | | | |
| --- | --- | --- | --- | --- | --- |
|  | AUC | 95%CI | Cut-off Point | Sensitivity (%) | Specificity (%) |
| **TyG index** | 0.679 | 0.676-0.682 | 8.42 | 67.5 | 59.5 |
| **TyG-BMI** | 0.720 | 0.717-0.723 | 177.08 | 88.2 | 38.9 |
| **TG/HDL-c** | 0.657 | 0.654-0.661 | 2.02 | 64.2 | 59.3 |
| **METS-IR** | 0.707 | 0.704-0.710 | 29.69 | 89.2 | 34.3 |
| **TyG index+Age** | 0.752 | 0.749-0.755 | 0.131* | 70.2 | 68.1 |
| **TG/HDL-c+Age** | 0.749 | 0.746-0.752 | 0.134* | 68.2 | 69.7 |
| **TyG-BMI+ Age** | 0.775 | 0.773-0.778 | 0.120* | 78.3 | 63.6 |
| **METS-IR+ Age** | 0.774 | 0.771-0.777 | 0.134* | 73.4 | 68.2 |

TG/HDL-c, triglycerides/high-density lipoprotein cholesterol ratio. TyG index, Triglyceride-glucose index. TyG-BMI, TyG index with body mass index. METS-IR, metabolic score for insulin resistance. AUC, area under the curve.

*refers to the predicted probability calculated by logistic regression.

***Supplementary Figure 1***. **Bayesian network model of hypertension prevalence and all clinical characteristics after multiple imputation.**


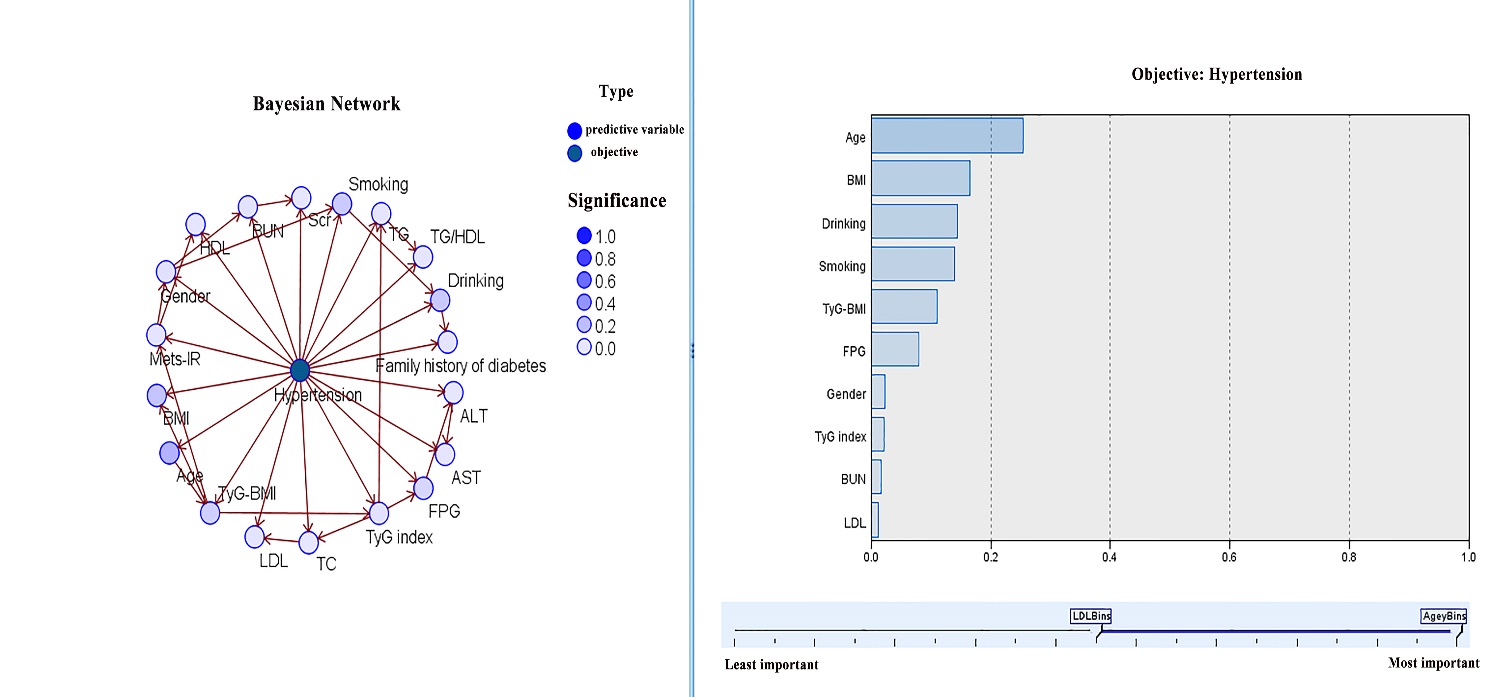


***Supplementary Figure 2.*** **Bayesian network model of hypertension prevalence and four IR surrogates-related characteristics after multiple imputation**


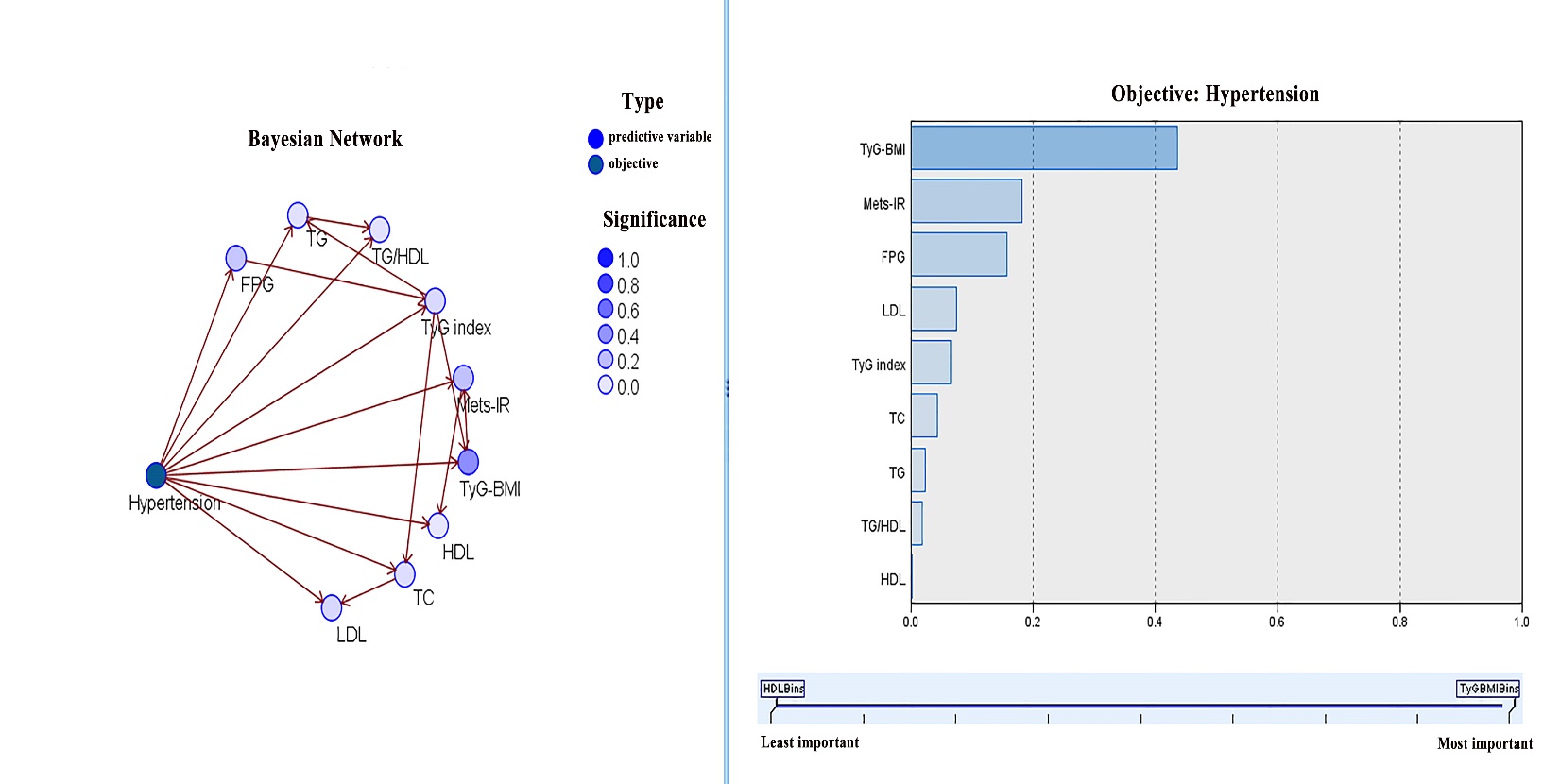

Supplement: Supplementary file 1 — Additional file1:Table S1. Baseline information of the overall population after multiple imputation *.Table S2. Multivariable logistic regression of four IR surrogates and the prevalence of hypertension after multiple imputation. Table S3. Predictive value of four IR substitutes for the prevalence of hypertension after multiple imputation. Figure S1. Bayesian network model of hypertension prevalence and all clinical characteristics after multiple imputation. Figure S2. Bayesian network model of hypertension prevalence and four IR surrogates-related characteristics after multiple imputation. [file 13098_2022_907_MOESM1_ESM.docx]
